# Assessment of dental pain and its association with dental anxiety and oral health-related quality of life

**DOI:** 10.2340/aos.v85.45322

**Published:** 2026-03-27

**Authors:** Azhar Iqbal, Osama Khattak, Yasir Dilshad Siddiqui, Nisreen Nabiel Hassan, Rital Jamal Alwaqid, Shahzad Ahmad, Muhammad Amber Fareed, Malik Adeel Anwar, Rakhi Issrani

**Affiliations:** a Department of Restorative Dental Sciences, College of Dentistry, Jouf University, Sakaka, Kingdom of Saudi Arabia; b Department of Preventive Dental Sciences, College of Dentistry, Jouf University, Sakaka, Kingdom of Saudi Arabia; c Department of Restorative Dental Sciences, Taibah University, Madina, Kingdom of Saudi Arabia; d Faculty of Medicine and Health Science, The University of Buckingham, Buckingham, United Kingdom; e Clinical Sciences Department, College of Dentistry, Ajman University, Ajman, United Arab Emirates; f Centre of Medical and Bio-allied Health Sciences Research, Ajman University, Ajman, United Arab Emirates; g Department of Oral Pathology, University College of Dentistry, The University of Lahore, Lahore, Pakistan

**Keywords:** Oral, quality of life, pain, fear, dental, survey

## Abstract

**Background:**

Dental anxiety (DA) and dental pain are closely interrelated. Anxiety often leads to an exaggerated perception of pain, while the experience of pain can further intensify anxiety, creating a vicious cycle. This cycle can significantly have an effect on individual’s oral health-related quality of life (OHRQoL). Therefore, it is essential to implement both clinical and psychological interventions to effectively manage DA.

**Aim:**

The aim of this study is to assess the level of dental pain and its association with DA and OHRQoL among patients attending dental treatment.

**Methodology:**

A cross-sectional study involving 805 participants was conducted using consecutive sampling. Data were collected on demographic characteristics, dental health status, and self-rated health. The participants’ dental pain and DA were assessed using a 100-mm Visual Analog Scale (VAS), the Dental Fear Survey (DFS), and the Dental Anxiety Scale (DAS). Descriptive analyses were performed, followed by logistic regression to identify determinants of Oral Impacts on Daily Performance (OIDP).

**Results:**

The study sample consisted of 805 participants, comprising 69.7% males and 30.3% females. Most participants were aged between 31 and 50 years (46.9%). The study’s findings revealed no significant differences in dental pain prevalence by gender, age, or education level. However, participants with dental pain reported greater difficulties in daily activities, particularly in eating and social interactions. Significant mean differences were observed in DA (*P* < 0.001) and DFS (*P* = 0.009), with individuals experiencing dental pain reporting higher mean scores for DA (7.74 vs. 7.10) and DFS (37.56 vs. 35.55). Logistic regression identified decayed teeth [OR = 1.48 (95% CI: 1.06, 2.05), *p* = 0.020] and higher dental pain levels [OR = 6.08 (95% CI: 4.39, 8.41), *p* < 0.001] as factors significantly associated with OIDP.

**Conclusion:**

The study shows a significant association between dental pain and a poor OHRQoL. These findings emphasize the importance of addressing dental health issues to improve overall well-being and quality of life among affected individuals.

## Introduction

Dental pain is an orofacial discomfort originating from the teeth and surrounding structures [1, 2]. Due to its high prevalence, it is recognized as a significant public health concern [3, 4]. The overall prevalence of dental pain varies widely, ranging from 17% to 71% depending on factors such as government policies, demographics, regions, and measurement methods [5–7]. Procedural pain, often associated with dental treatments, has been extensively validated in the literature [7]. Studies indicate that dental anxiety (DA) consistently influences pain perception prior to dental procedures [8]. Svensson et al. highlighted that DA is a common health issue that impacts both individuals and society [9]. Its prevalence varies across cultures and countries, with rates ranging from 4% to 20% [10–13]. Oral health-related quality of life (OHRQoL) is affected by three main factors: pain and discomfort, functionality, and appearance [9, 10]. Conversely, DA is linked to poorer oral health, functional impairments, and avoidance of dental care [9, 14–16]. Recent research has established a connection between DA and lower OHRQoL [17–19], with a correlation between the degree of impairment and the level of DA [9, 20].

Being a vital aspect of the overall health and well-being, OHRQoL has significant implications in dental research and clinical practice [5, 21, 22]. The use of different OHRQoL measurement tools enables the clinicians to assess the extent to which various aspects of life of the individuals and their families have been impacted by oral health issues [5, 22, 23]. These assessments can address factors such as social interaction, emotional well-being, self-image, psychological effects, and functionality [5, 22–24]. Managing patients with severe dental pain presents challenges for dental professionals, particularly when these patients exhibit behaviors associated with missed appointments, poor oral hygiene, and high levels of DA [25, 26].

Understanding the interplay between dental pain and OHRQoL is essential for improving dental care standards, particularly among individuals with DA. According to Wilson and Cleary’s model of health-related quality of life [27], clinical symptoms such as acute dental pain influence functional status and perceived well-being through physiological, psychological, and behavioral pathways. Similarly, Lazarus and Folkman’s Transactional Model of Stress and Coping suggests that individuals’ cognitive and emotional responses to pain, such as anxiety and perceived threat, mediate how they experience and adapt to oral health challenges [28]. Employing these frameworks, this study aims to assess the level of dental pain and its association with DA and OHRQoL among patients seeking dental treatment in the Jouf region of Saudi Arabia. By integrating these theoretical models, this study seeks to provide a more comprehensive understanding of how psychological and physical factors improve oral health outcomes. Figure 1 illustrates the directed acyclic graph (DAG) representing the conceptual framework of the study’s aim.

**Figure 1:**
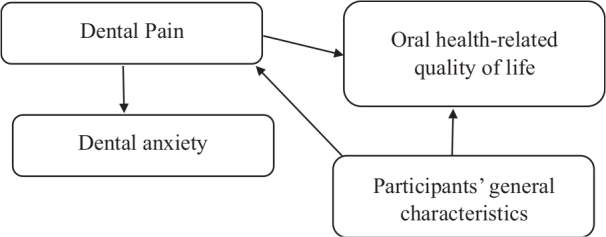
Directed acyclic graph (DAG) representing the conceptual framework of the study.

## Methods and materials

### Study design

This study employed a cross-sectional design and included a successive sample of 805 adults experiencing dental pain of varying intensity and DA. Data collection took place between May 15, 2024 and September 15, 2024.

### Sample size estimation

Sample size determination was conducted using G*Power version 3.1.9.4. The initial estimate indicated a need for 786 participants, based on an anticipated proportion of 0.05, a small effect size, a margin of error of 0.05, and a study power of 80%. To account for a potential 5% dropout rate, the final corrected sample size was adjusted to 827 to accommodate possible missing data and entry errors.

### Study setting

The study’s was conducted in the university dental clinics of the College of Dentistry, Jouf University, a public sector university, in the Jouf region, located in the north of Saudi Arabia. Being a public sector dental institute, the dental services are provided to the population of this region free of cost. In the university dental clinics, the oral medicine clinics are available, providing the consultation and assessment of the patients presenting with DA.

### Inclusion and exclusion criteria

The inclusion criteria consisted of individuals, who are 20 years of age or older and have refused to undergo conventional dental treatment. Those with significant mental health disorders were excluded from the study. Ultimately, the final sample consisted of 805 individuals, of whom 561 (69.7%) were male and 244 (30.3%) were female. Informed consent was obtained from all participants after they were informed of the study’s purpose. The ethical approval for this study was obtained from the Local Committee of Bioethics (approval number 1-09-45).

### Procedure

#### Calibration of the examiners

Before the start of data collection, the three participating dentists underwent a process of calibration to ensure consistency while interpreting panoramic radiographs and conducting clinical examinations. The calibration process involved reviewing a standardized set of 30 panoramic radiographs, which were representing a wide range of common dental pathologies leading to acute dental pain, for example, caries and apical periodontitis. Each participating dentist assessed the radiographs independently, and the findings were compared against a standard reference established by a senior endodontist with over 15 years of experience. The inter-examiner reliability was measured using a statistical method, Cohen’s kappa, requiring a minimum kappa value of 0.8 before commencing the data collection. The consensus meetings were carried out, including the participating dentists and a senior endodontist with over 15 years of experience, to discuss the discrepancies until uniform interpretation criteria to collect the data were agreed upon. This calibration process helped to reduce diagnostic variability and to improve inter-examiner reliability during radiographic assessments throughout the study.

### Study settings

The study was conducted in university dental clinics, college of dentistry, Jouf University, a public sector dental institute, providing the dental services free of cost to the population of Jouf region of Saudi Arabia. In the university dental clinics, the oral medicine clinics are available providing consultation and assessment for the patients presenting with DA.

### Recruitment of the participants

Participants were recruited from the university dental clinics of the college of dentistry, Jouf University. The recruitment was done using consecutive sampling of the participants reporting to the dental clinics with acute dental pain. They were approached for participation in this study after taking and signing an informed consent.

At the initial visit, all participants completed questionnaires that assessed their general characteristics (including gender, age, education, reasons for dental visits, and region) and DA status, following the inclusion criteria. Participants who consented to further involvement underwent a structured interview with the researcher, which included additional questionnaires addressing perceived oral health, general health ratings, dental pain status, and OHRQoL. Clinical findings and characteristics were recorded during the second and third clinic appointments each of which was taking place after a week interval, utilizing dental pantomogram and a modified clinical examination.

Three dentists participated in the examination process, and a consensus was reached to calibrate the examiners based on the findings from the panoramic radiographs and the modified clinical examinations, minimizing the risk of diagnostic errors.

### Study instruments

A structured questionnaire consisting of eight items was used to obtain information on dental pain experiences across different orofacial regions. Participants completed the questionnaire through an interview administered by a participating dentist. The study consisted of three key questions on dental pain: (1) ‘Did you have a toothache during the past month?’ (2) ‘Did you feel pain when eating or drinking hot and/or cold food/beverages during the past month?’ and (3) ‘Did you feel pain when chewing food during the past month?’ Response options were ‘yes’ or ‘no’, For participants who answered ‘yes’, pain intensity was measured using a 100-mm Visual Analog Scale (VAS), with 0 representing no pain and 100 representing extreme pain. Based on these items, a dichotomous dental pain index was created, classifying individuals as experiencing dental pain if they responded ‘yes’ to at least one of the three questions [9]. This index was then applied in the subsequent analyses.

The Oral Impacts on Daily Performance Index (OIDP) was used to assess OHRQoL [29]. The OIDP scale consists of nine items covering three domains – physical, psychological, and social performance – related to difficulties with the mouth or teeth over the preceding 6 months. Participants who responded ‘yes’ to any item rated the impact using two dimensions: frequency and severity. Frequency scores: 5 = every day or nearly every day, or more than 3 months in total; 4 = three to four times a week, or up to 3 months; 3 = once or twice a week, or up to 30 days; 2 = once or twice a month, or up to 15 days; 1 = less than once a month, or up to 5 days; 0 = no days, no impact. Severity scores: 0 = no effect, 1 = slight effect, 2 = modest effect, 3 = moderate effect, 4 = severe effect, 5 = extremely severe effect. For each item, the frequency score was multiplied by the severity score to obtain an impact score. With nine items, the maximum possible score was 225. Total scores were standardized as percentages by dividing by the maximum score and multiplying by 100, allowing comparisons across individuals. Additionally, the OIDP prevalence score (range 0–9) was calculated to identify whether participants experienced at least one impact on daily activities. This was recoded into a binary outcome: 0 = no daily activity affected and 1 = at least one daily activity affected. The validity of the OIDP prevalence score has been confirmed in a previous study [29].

DA was assessed using two self-report instruments: the Dental Fear Survey (DFS) and the Dental Anxiety Scale (DAS) [26, 30]. The DFS [26] consists of 20 items that evaluate anticipatory anxiety, avoidance behavior, physiological arousal, and fear of specific dental stimuli. Each item is rated on a scale from 1 (no anxiety) to 5 (high anxiety), resulting in a total score ranging from 20 to 100, with higher scores indicating greater levels of DA. A total score of 60 or above is commonly used as the threshold for identifying DA [26]. The DAS [30] includes four items that describe imagined dental conditions, each rated on a scale from 1 (no anxiety) to 5 (extreme anxiety). The total score ranges from 4 to 20, with higher scores reflecting greater anxiety. A score of 13 or above is considered indicative of severe DA [30].

Self-rated oral and general health was assessed using two single-item questions: ‘How do you rate your overall oral health?’ and ‘How do you rate your overall general health?’ Responses were recorded on a 100-mm VAS, ranging from 0 (worst possible) to 100 (best possible).

Clinical status was evaluated by recording the total number of missing, decayed, filled, and root-filled teeth, as well as teeth with apical periodontitis, excluding third molars. For subsequent analyses, outcomes were dichotomized as follows: number of decayed teeth (0 = ≤ 1, 1 ≥ 1), and number of missing teeth, root-filled teeth, and teeth with apical periodontitis (0 = none, 1 ≥ 1).

The study questionnaires were adapted and modified from a previous study [26, 29, 30]. Since they had not been previously tested or validated in this specific population, content validity was established by consulting six experts in dental surgery. These experts evaluated the relevance of each item to its corresponding construct through a face-to-face review to ensure the questionnaire accurately measured its intended content. Minor revisions were made to certain items to enhance their cultural appropriateness and clarity for the target participants. The content validity was assessed by calculating the item-level content validity index (I-CVI) and the scale-level content validity index (S-CVI), both of which yielded values of 0.83.

### Data analysis

Statistical analyses were carried out using Statistical Product and Service Solutions (SPSS) version 29. The procedures included descriptive statistics, Pearson’s chi-square tests, independent t-tests, and binary logistic regression. Descriptive statistics summarized the data in terms of means, standard deviations, frequencies, and percentages. Pearson’s chi-square tests were applied to examine associations between participants’ characteristics and dental pain status, differences in the prevalence of dental pain between males and females, and differences in OIDP prevalence and frequency across different dental pain statuses. Independent t-tests were used to assess mean differences in DA, DFS, and OIDP, across dental pain status; DA and DFS across OIDP; and dental pain scores between males and females.

Furthermore, binary logistic regression was employed to identify significant determinants of OIDP. Multiple logistic regression analyses were conducted using both forward LR and backward LR methods, with the final model established using the enter method. The adequacy of the final model was evaluated using several criteria, including assessment of variable interactions, the Hosmer–Lemeshow goodness-of-fit test, classification accuracy, and the area under the receiver operating characteristic (ROC) curve (AUC). The adjusted odds ratios (ORs) were reported together with their corresponding 95% confidence intervals (CIs). A *p*-value of < 0.05 was considered statistically significant.

## Results

Table 1 presents the general characteristics of the study participants and their association with dental pain. Most participants were male (69.7%), with the largest age group being 41–50 years (25.8%). Nearly half held a university degree (49.8%). A considerable proportion reported visiting a dental clinic due to pain (28.8%), and the highest regional representation was from the northern region (36.3%). In terms of oral health status, the majority had no missing teeth (56.9%), no root remnants (65.0%), decayed teeth (61.9%), filled teeth (56.0%), no root-filled teeth (66.8%), and no apical periodontitis (88.9%). The mean self-rated oral health score was 2.32 (standard deviation [SD] = 0.74), while self-rated overall health was 2.23 (SD = 0.88).

**Table 1:** General characteristics of study participants and their association with dental pain status (N = 805).

Variable	Total	No dental pain (*n* = 334)	Dental pain (*n* = 471)	*p*-value*
Gender [*n* (%)]				0.784
Male	561 (69.7)	231 (41.2)	330 (58.8)	
Female	244 (30.3)	103 (42.2)	141 (57.8)	
Age [*n* (%)]				0.162
20–30	129 (16.0)	56 (43.4)	73 (56.6)	
31–40	170 (21.1)	76 (44.7)	94 (55.3)	
41–50	208 (25.8)	70 (33.7)	138 (66.3)	
51–60	169 (21.0)	70 (41.4)	99 (58.6)	
61–70	77 (9.6)	36 (46.8)	41 (53.2)	
71–80	44 (5.5)	21 (47.7)	23 (52.3)	
81–90	8 (1.0)	5 (62.5)	3 (37.5)	
Education [*n* (%)]				0.272
Elementary	126 (15.7)	45 (35.7)	81 (64.3)	
High school	278 (34.5)	123 (44.2)	155 (55.8)	
University	401 (49.8)	166 (41.4)	235 (58.6)	
Reasons to visit dentist [*n* (%)]				0.457
Routine checkup	217 (27.0)	91 (41.9)	126 (58.1)	
Pain	232 (28.8)	93 (40.1)	139 (59.9)	
Swelling	193 (24.0)	73 (37.8)	120 (62.2)	
Filling	97 (12.0)	47 (48.5)	50 (51.5)	
Teeth cleaning	66 (8.2)	30 (45.5)	36 (54.5)	
Region [*n* (%)]				0.924
East	103 (12.8)	43 (41.7)	60 (58.3)	
West	133 (16.5)	57 (42.9)	76 (57.1)	
North	292 (36.3)	116 (39.7)	176 (60.3)	
South	117 (14.5)	52 (44.4)	65 (55.6)	
Central	160 (19.9)	66 (41.3)	94 (58.8)	
Self-rated oral health [Mean (SD)]	2.32 (0.74)	2.34 (0.75)	2.30 (0.74)	0.481^+^
Self-rated general health [Mean (SD)]	2.11 (0.93)	2.08 (0.93)	2.12 (0.93)	0.545^+^
Self-rated overall health [Mean (SD)]	2.23 (0.88)	2.22 (0.89)	2.23 (0.88)	0.818^+^
Missing [*n* (%)]				0.388
No	458 (56.9)	196 (42.8)	262 (57.2)	
Yes	347 (43.1)	138 (39.8)	209 (60.2)	
Root remnants [*n* (%)]				0.369
No	523 (65.0)	211 (40.3)	312 (59.7)	
Yes	282 (35.0)	123 (43.6)	159 (56.4)	
Decayed teeth [*n* (%)]				0.262
No	307 (38.1)	135 (44.0)	172 (56.0)	
Yes	498 (61.9)	199 (40.0)	299 (60.0)	
Filled teeth [*n* (%)]				0.653
No	354 (44.0)	150 (42.4)	204 (57.6)	
Yes	451 (56.0)	184 (40.8)	267 (59.2)	
Root filled teeth [*n* (%)]				0.906
No	538 (66.8)	224 (41.6)	314 (58.4)	
Yes	267 (33.2)	110 (41.2)	157 (58.8)	
Apical periodontitis [*n* (%)]				0.261
No	716 (88.9)	302 (42.2)	414 (57.8)	
Yes	89 (11.1)	32 (36.0)	57 (64.0)	
DA [Mean (SD)]	7.40 (2.48)	7.10 (2.46)	7.74 (2.46)	< 0.001^+^
DFS [Mean (SD)]	36.66 (9.23)	35.55 (9.07)	37.56 (9.33)	0.009^+^
OIDP	1.84 (1.94)	1.06 (1.45)	2.70 (2.04)	< 0.001^+^

* Pearson’s chi-square test,

+ Independent *t*-test.

SD: standard deviation; DA: dental anxiety; DFS: Dental Fear Survey; OIDP, Oral Impacts on Daily Performance.

The prevalence of dental pain was higher among males (58.8%), participants aged 41–50 years (66.3%), those with elementary education (64.3%), individuals who visited the dentist due to swelling (62.2%), and participants from the northern region (60.3%). Higher prevalence was also recorded among participants with missing teeth (60.2%), no root remnants (59.7%), decayed teeth (60.0%), filled teeth (59.2%), root-filled teeth (58.8%), and apical periodontitis (64.0%). Significant mean differences were observed in DA (*P* < 0.001), DFS (*P* = 0.009), and OIDP (*P* < 0.001), with individuals experiencing dental pain reporting higher mean scores for DA (7.74), DFS (37.56), and OIDP (2.70).

Table 2 shows the association between participants’ general and clinical characteristics and OIDP. The findings revealed that only decayed teeth (*P* = 0.010) and DFS (*P* = 0.040) were significantly associated with OIDP. Participants who reported at least one daily activity affected were more likely to have decayed teeth (70.7%) and a higher mean DFS score (37.13 vs. 35.70).

**Table 2:** General characteristics of study participants and their association with OIDP (N = 805).

Variable	No daily activity affected (*n* = 263)	At least one daily activity affected (*n* = 542)	*p*-value*
Gender [*n* (%)]			0.709
Male	181 (32.3)	380 (67.7)	
Female	82 (33.6)	162 (66.4)	
Age [*n* (%)]			0.050
20–30	54 (41.9)	75 (58.1)	
31–40	60 (35.3)	110 (64.7)	
41–50	52 (25.0)	156 (75.0)	
51–60	50 (29.6)	119 (70.4)	
61–70	28 (36.4)	49 (63.6)	
71–80	16 (36.4)	28 (63.6)	
81–90	3 (37.5)	5 (62.5)	
Education [*n* (%)]			0.141
Elementary	41 (32.5)	85 (67.5)	
High school	79 (28.4)	199 (71.6)	
University	143 (35.7)	258 (64.3)	
Reasons to visit dentist [*n* (%)]			0.146
Routine checkup	74 (34.1)	143 (65.9)	
Pain	81 (34.9)	151 (65.1)	
Swelling	49 (25.4)	144 (74.6)	
Filling	33 (34.0)	64 (66.0)	
Teeth cleaning	26 (39.4)	40 (60.6)	
Region [*n* (%)]			0.470
East	41 (39.8)	62 (60.2)	
West	46 (34.6)	87 (65.4)	
North	89 (30.5)	203 (69.5)	
South	38 (32.5)	79 (67.5)	
Central	49 (30.6)	111 (69.4)	
Self-rated oral health [Mean (SD)]	2.30 (0.74)	2.33 (0.74)	0.591^+^
Self-rated general health [Mean (SD)]	2.07 (0.92)	2.12 (0.94)	0.430^+^
Self-rated overall health [Mean (SD)]	2.24 (0.92)	2.22 (0.87)	0.763^+^
Missing [*n* (%)]			0.482
No	145 (31.7)	313 (68.3)	
Yes	118 (34.0)	229 (66.0)	
Root remnants [*n* (%)]			0.215
No	163 (31.2)	360 (68.8)	
Yes	100 (35.5)	182 (64.5)	
Decayed teeth [*n* (%)]			**0.010**
No	117 (38.1)	190 (61.9)	
Yes	146 (29.3)	352 (70.7)	
Filled teeth [*n* (%)]			0.086
No	127 (35.9)	227 (64.1)	
Yes	136 (30.2)	315 (69.8)	
Root filled teeth [*n* (%)]			0.778
No	174 (32.3)	364 (67.7)	
Yes	89 (33.3)	178 (66.7)	
Apical periodontitis [*n* (%)]			0.224
No	239 (33.4)	477 (66.6)	
Yes	24 (27.0)	65 (73.0)	
DA [Mean (SD)]	7.33 (2.57)	7.44 (2.43)	0.561^+^
DFS [Mean (SD)]	35.70 (9.24)	37.13 (9.20)	**0.040** ^+^

* Pearson’s chi-square test,

+ Independent *t*-test.

SD: standard deviation; DA: dental anxiety; DFS: Dental Fear Survey.

The prevalence of having at least one daily activity affected was greater among males (67.7%), those aged 41–50 years (75.0%), participants with high school education (71.6%), individuals who visited the dentist due to swelling (74.6%), and those residing in the northern region (69.5%). OIDP was also more common among participants with no missing teeth (68.3%), no root remnants (68.8%), decayed teeth (70.7%), filled teeth (69.8%), no root-filled teeth (67.7%), apical periodontitis (73.0%), and those with higher mean DA scores (7.44 vs. 7.33).

Table 3 presents the prevalence and frequency of OIDP scores for the study participants across dental pain status. The findings show that participants with OHRQoL issues reported significantly higher levels of dental pain across all OIDP items (*P* < 0.05), except for difficulties with speech or word articulation (*P* = 0.693). Interestingly, those who reported speech or word difficulties still had a higher prevalence of dental pain (61.4%) compared to those without such difficulties (58.3%).

**Table 3:** The prevalence of OIDP items and their association with dental pain status.

Statement	Total (*N* = 805) [*n* (%)]	Presence of dental pain (*N* = 334) [*n* (%)]	Absence of dental pain (*N* = 471) [*n* (%)]	*p*-value*
Are you having difficulties in eating (biting, chewing)?				**0.005, S**
No	663 (82.4)	290 (43.7)	373 (56.3)	
Yes	142 (17.6)	44 (31.0)	98 (69.0)	
Are you having difficulties with speech or word difficulties?				0.693
No	761 (94.5)	317 (41.7)	444 (58.3)	
Yes	44 (5.5)	17 (38.6)	27 (61.4)	
Are you having problems washing your mouth due to mouth-related issues?				**0.022, S**
No	713 (88.6)	306 (42.9)	407 (57.1)	
Yes	92 (11.4)	28 (30.4)	64 (69.6)	
Have you had sleepless nights due to toothaches or other mouth related issues?				**< 0.001, S**
No	589 (73.2)	286 (48.6)	303 (51.4)	
Yes	216 (26.8)	48 (22.2)	168 (77.8)	
Did you feel ill because of problems inside your mouth?				**< 0.001, S**
No	563 (69.9)	289 (51.3)	274 (48.7)	
Yes	242 (30.1)	45 (18.6)	197 (81.4)	
Do you avoid smiling or showing your teeth because of problems inside your mouth?				**< 0.001, S**
No	680 (84.5)	315 (46.3)	365 (53.7)	
Yes	125 (15.5)	19 (15.2)	106 (84.8)	
Have toothaches or other mouth- related problems ever prevented you from attending schools?				**< 0.001, S**
No	590 (73.3)	286 (48.5)	304 (51.5)	
Yes	215 (26.7)	48 (22.3)	167 (77.7)	
Have your problems with teeth prevented you from meeting your friends or from other social activities?				**< 0.001, S**
No	540 (67.08)	264 (48.9)	276 (51.1)	
Yes	265 (32.91)	70 (26.4)	195 (73.6)	

* Pearson’s chi-square test, *S = Significant*.

Table 4 displays the results of the binary logistic regression analysis. After adjusting for participants’ general characteristics, the final model identified two factors that are significantly associated with OIDP: the dental pain index and the number of decayed teeth. Specifically, individuals who reported higher dental pain levels were over six times more likely to experience adverse impacts on their daily activities related to oral health (OR = 6.08, 95% CI: 4.39–8.41, *P* < 0.001). This finding underscores the strong influence of pain intensity on functional and psychosocial aspects of oral well-being. Similarly, participants with more decayed teeth had a 1.48 times increased likelihood of reporting oral health-related impacts (OR = 1.48, 95% CI: 1.06–2.05, *P* = 0.020). Overall, these results highlight that both symptomatic (pain-related) and clinical (tooth decay) indicators are critical determinants of oral health’s quality of life, suggesting that interventions aimed at preventing and managing dental decay and pain reduce the burden of OIDP among affected individuals.

**Table 4:** The factors associated with OIDP (no daily performance versus one or more daily performance reported) using multiple logistic regression analyses.

Variable	Referent category	Category	OR	95% CI	*p*-value*
Decayed teeth	No pain	Pain	1.48	1.06, 2.05	0.020
Dental pain	≤ 1	> 1	6.08	4.39, 8.41	< 0.001

* Binary logistic regression.

OR: odds ratio; CI: confidence interval.

The model demonstrated an adequate fit, as shown by the Hosmer–Lemeshow test (*P* = 0.611), indicating that the observed data were consistent with the predicted probabilities. The satisfactory classification accuracy was 71.6%, indicating the percentage of correctly distinguishing between individuals with and without OIDP. Also, the area under the receiver operating characteristic curve (AUC) was 72.9% (Figure 1), reflecting good discriminative ability. This implies that the model was able to correctly classify cases approximately 73% of the time, indicating a reliable balance between sensitivity and specificity in predicting OIDP outcomes.

**Figure 1:**
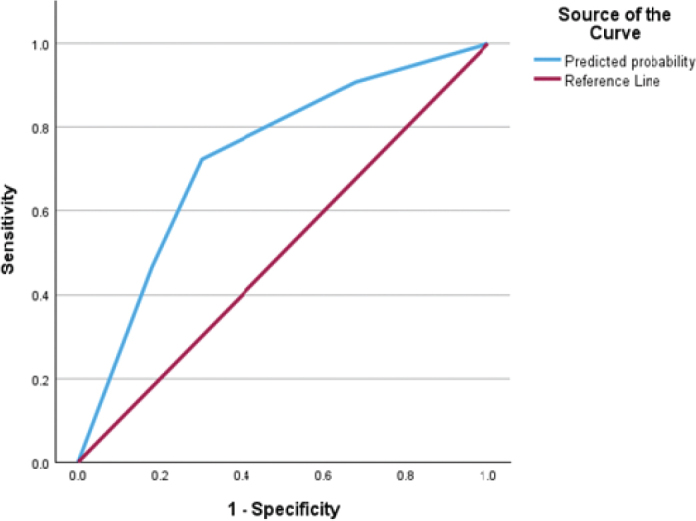
Receiver operating characteristics (ROC) curve of the final logistic regression model of OIDP.

## Discussion

This study to assess the level of dental pain and its association with DA and OHRQoL among individuals requiring dental treatment across different regions of Saudi Arabia. Pain is a complex and subjective experience that varies considerably among individuals. Although self-reported measures are commonly used, instruments such as the McGill Pain Questionnaire provide qualitative assessments of pain [31], though their length can limit practicality in clinical settings [32]. In this study, we focused specifically on participant-reported dental pain, excluding pain related to dental procedures. The survey instruments employed had been previously validated for reliability and accuracy and have also been applied in other studies [33, 34].

The findings of our study showed no strong overall association between dental pain and participants’ sociodemographic characteristics. However, dental pain was more prevalent among individuals presenting with certain clinical conditions, including apical periodontitis, decayed teeth, filled teeth, root-filled teeth, and missing teeth, as well as among those without root remnants. This suggests that dental pain may be more closely linked to pathological or restorative dental conditions rather than to general demographic factors. Additionally, the higher prevalence of dental pain among participants with DA highlights the complicated interaction between psychological factors and pain perception. Individuals with DA may exhibit heightened sensitivity to oral symptoms, leading to greater reporting of pain. These findings are consistent with previous research indicating that both clinical oral health status and psychological factors contribute significantly to the experience and reporting of dental pain [3, 5, 7].

The results indicate that decayed teeth and dental fear (DFS) were the only significant determinants of OIDP. Participants who reported at least one daily activity affected by oral conditions were significantly more likely to have decayed teeth, with 70.7% presenting at least one untreated caries lesion. This finding highlights the critical effect of untreated dental caries on everyday functioning, including eating, speaking, and social interactions. A possible explanation is the vicious cycle in which fear of dental treatment discourages timely care-seeking, leading to deterioration in oral health and, consequently, greater disruptions in daily life [9]. Consistent with the findings of Svensson et al. [9], no other variables demonstrated a statistically significant association with OIDP. This suggests that, in this population, clinical status (decayed teeth) and psychological factors (dental fear) have a greater impact on OHRQoL than demographic or general health characteristics. These results highlight the importance of dental care strategies that address both disease management and psychological barriers to treatment to improve patients’ daily functioning and overall well-being.

The overall prevalence of DA in this study, as calculated by the DFS and DAS, was found to be 1.7% and 1.9%, respectively. The findings suggest that DA, DFS, OIDP are key psychological factors associated with the experience of dental pain. Participants with dental pain had slightly higher mean DA (7.74), DFS (34,35), and OIDP (2.70) scores, emphasizing the idea that emotional and psychological distress may heighten pain perception or influence how individuals interpret oral discomfort. In contrast, none of the other general characteristics, such as age, gender, self-rated health, or other sociodemographic variables, showed a significant association with dental pain. This suggests that psychological factors may be more predictive of dental pain experiences than demographic or general health indicators in this sample. Recent literature suggests that individuals experiencing DA often have poorer oral health, characterized by a higher number of decayed teeth, teeth affected by apical periodontitis, and the presence of root remnants [9]. These observations align with numerous previous studies [35–37].

Over half of the participants reported a history of dental pain, with nearly 50% experiencing all three types of pain assessed. The types of pain included: (1) any dental pain (with or without provocation), (2) moderate-to-severe tooth sensitivity when chewing hot food, and (3) moderate-to-severe pain when exposed to cold stimuli. Previous population-based surveys have reported that up to 30% of adults experience tooth discomfort [9, 33, 34, 38]. Interestingly, more than twice as many men reported recent pain from hot or cold foods and drinks compared to women, which contrasts with Svensson et al., who found that women reported more pain in similar situations [9].

Regarding OIDP scores, participants with poorer OHRQoL exhibited significantly higher scores across all items, except for difficulties in speaking or pronouncing words. Notably, individuals who had trouble speaking or pronouncing words, regardless of age, reported experiencing dental pain more frequently than those without such difficulties. Participants with tooth pain also demonstrated reduced OHRQoL, as indicated by both the OIDP prevalence score and summary index score. These results are consistent with the findings of Svensson et al. who established significant association between OHRQoL and oral pain in patients with severe DA [9]. Moreover, studies by Carlsson et al. [17], Heidari et al. [19] and Boman et al. [29] identified a strong connection between poor OHRQoL and dental pain.

This study highlighted that the dental pain index and the presence of decayed teeth were significant determinants of at least one impact on the OIDP. This supports recent research identifying dental pain and decayed teeth as key determinants of low OHRQoL [9]. A number of studies in the literature also corroborate the link between lower OHRQoL and dental pain [5, 6, 39–41]. Considering the findings from various studies that indicate a correlation between the frequency and severity of dental pain and poor OHRQoL, it is essential to provide evidence-based dental care that adheres to best practices. This approach can help to break the cycle of pain and anxiety, ultimately improving OHRQoL [5].

This study underscores the urgency of therapeutic and preventive measures to mitigate the detrimental effects of dental pain on daily activities and overall quality of life. Previous research supports this, indicating that untreated dental pain can lead to significant psychological repercussions [1, 21]. For children and adolescents, dental pain not only affects their learning and daily tasks but also impacts chewing, sleep, and social-emotional well-being [21, 40–42]. Furthermore, the effects extend to families, who may face missed workdays and the financial burden of treatment [3, 43]. Parents often experience anxiety and guilt related to their children’s dental health issues [21, 40].

Despite its contributions, this study has limitations. The assessment of oral conditions relied on panoramic radiographs and specific clinical tests, which may have led to an underestimation of oral disorders. The cross-sectional design restricts the interpretation of findings to associations rather than causal relationships, and the lack of a control group limits certain aspects of the results. However, the study incorporated both clinical indicators of oral disorders and established questionnaires, such as the OIDP, DAS, and DFS, alongside previously validated questions regarding tooth pain. Additionally, it included a substantial sample from diverse regions of Saudi Arabia, enhancing the generalizability of the findings.

## Conclusion

This study revealed that dental pain was prevalent among adults, with males reporting higher pain intensity than females. Furthermore, a significant association was found between dental pain and poor OHRQoL. To strengthen the evidence base and improve the findings of this study, future research should utilize more rigorous study designs, such as longitudinal and randomized trials. These studies should include representative samples, enforce strict eligibility criteria, and better control for confounding factors.
